# Gene Size Matters

**DOI:** 10.1371/journal.pone.0049093

**Published:** 2012-11-09

**Authors:** Alexandra Mirina, Gil Atzmon, Kenny Ye, Aviv Bergman

**Affiliations:** 1 Department of Systems and Computational Biology, Albert Einstein College of Medicine, Bronx, New York, United States of America; 2 Department of Genetics, Albert Einstein College of Medicine, Bronx, New York, United States of America; 3 Department of Medicine, Albert Einstein College of Medicine, Bronx, New York, United States of America; 4 Department of Epidemiology and Population Health, Albert Einstein College of Medicine, Bronx, New York, United States of America; University of Illinois at Chicago, United States of America

## Abstract

In this work we show that in genome-wide association studies (GWAS) there is a strong bias favoring of genes covered by larger numbers of SNPs. Thus, we state here that there is a need for correction for such bias when performing downstream gene-level analysis, e.g. pathway analysis and gene-set analysis. We investigate several methods of obtaining gene level statistical significance in GWAS, and compare their effectiveness in correcting such bias. We also propose a simple algorithm based on first order statistic that corrects such bias.

## Introduction

A large number of genome-wide association studies (GWAS) have been conducted in recent years. The purpose of such studies is screening for genetic markers that are associated with common diseases. The loci of genetic markers, and eventually genes at those loci, are further investigated on their roles in the etiology of disease, which may lead to a combination of improved diagnosis, treatment, and preventative measures.

In a typical GWAS, all subjects are genotyped at hundreds of thousands, and up to a couple of millions, DNA markers that are pre-selected to cover the entire genome. Usually, p-values are used to assess statistical significance at each DNA marker, and susceptible loci are identified where DNA markers have p-values lower than a specified threshold. The threshold is determined with the goal of controlling the number of expected false positives and adjusted for multiple testing. The genes that are in close proximity of the most significant loci are often treated to be the most relevant to the analyzed trait and are investigated in the downstream analysis. For example, in two recently published GWAS studies, Timmann et al. [1] suggests that ATP2B4 is related to severe malaria based on the fact that several associated SNPs are inside the genes; and Dunlop et al [2] suggested that CDKN1A, POLD3 and SHROOM2 are related to colorectal cancer as association are found in nearby SNP loci. We argue here that genes near the most significant SNP markers are not necessarily the most relevant to the disease in question and it is not always appropriate to select genes near the most significant markers for the purpose of gene-level analyses such as pathway analysis and gene-set analysis [3], [4]. *Since there is a large variation in number of markers covering each gene, this selection process based on p-value at individual markers is biased toward genes saturated with SNPs.* For example, consider a case-control study with 1,000 cases and 1,000 controls. Suppose gene A has a single SNP and that its frequency of a risk genotype is 5% in the control population and 6% in the case population. The power to detect the difference is 3×10^−5^ at the significance level of 10^−6^ [5] if the Pearson Chi-Squared test is used, and slightly higher if the G-test is used (4.3×10^−5^ according to our simulation). Now consider gene B, which has 100 SNPs but has no association with the disease. The probability of at least one of the 100 SNPs obtaining a p-value <10^−6^ is approximately 10^−4^. Thus, with a p-value cut-off at 10^−6^, Gene A is less likely to be selected than Gene B, even if the former is disease related and the latter is not.

Furthermore, if gene sets (pathways, functions etc.) are different in average genes sizes, then having a bias towards genes with larger number of SNPs in GWASs may result into subsequent bias of favoring pathways that relate to larger genes on average in subsequent gene set enrichment analysis.

## Results

### Bias in Reported GWAS Genes

**Figure 1:**
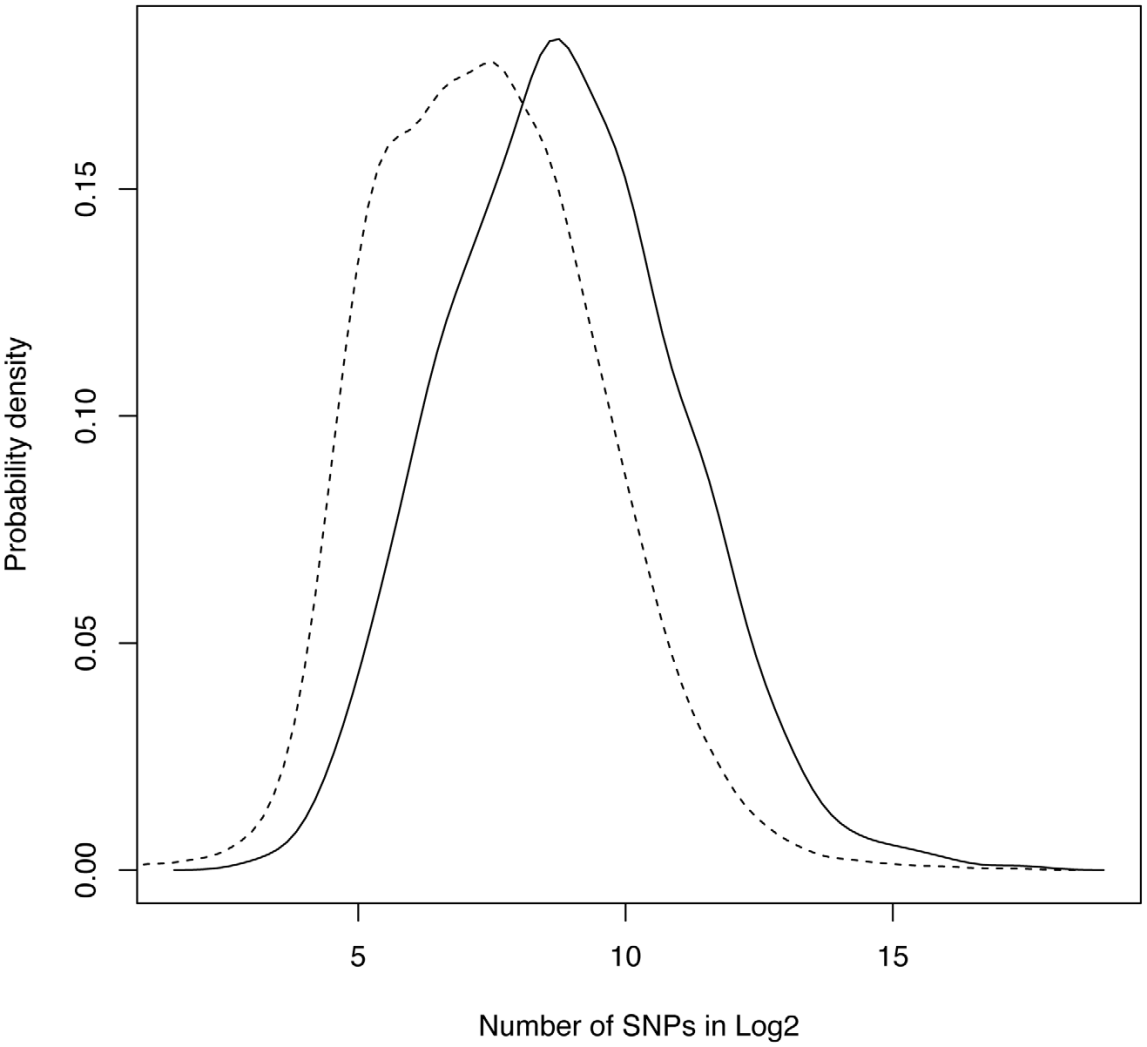
Distribution of the number of SNPs in log_2_. TRG – solid line, all genes – dashed line.

From the above arguments, we hypothesize that among genes reported by GWASs there is a bias favoring genes of large size as they usually contain more SNP markers. To verify our hypothesis, we examined 2,504 reported disease/trait related genes (TRGs) available from *A Catalog of Published Genome-Wide Association Studies* [6]. The catalog includes genes reported in more than 800 genome-wide associating studies; each used at least 100,000 SNP markers before filtering. Only those genes that has SNP markers with p-values <10^−5^ were ascertained in the database, resulting 4,736 implicated genes, of which 2,504 are unique. We compared the distribution of the number of SNPs in reported TRGs (solid line, Fig.1) to the distribution in all 26,125 genes annotated in the NCBI dbSNP [7] (dashed line, Fig. 1). It is clear that those reported TRGs tend to have more SNPs than the average of genes present in human genome. The average number of SNPs per reported TRG was 1,715, almost three times more than of all genes, which is 638. The comparison of the two distributions (Fig. 1) using the two-sample Kolmogorov-Smirnov test resulted in high statistical significance (p-value = 8.11×10^−144^), which indicates that there is indeed a notable bias in GWASs reporting genes containing a larger number of SNPs.

### Gene Size Differences Among Gene Sets

To examine if the gene size differs among gene groups, we obtained a list of Gene Ontology categories of the highest hierarchical level under the section of “Biological Process” [8] (Table 1). Out of 33 total classes, three (GO:0006794, GO:0006794, GO:0015976) had no associated genes in human, and two (GO:0019740, GO:0009758) had low number of genes (1 and 2 respectively), which left us with the remaining 28. Number of SNPs was assigned to each gene as described in the method section. One-way ANOVA is used to test the null hypothesis that mean SNP numbers per gene in each GO category are equal and p-value of the F-test is 1.88×10^−8^. This suggests that these gene sets are different in average genes sizes. In order to further investigate this issue we performed the same analysis on sets of categories of two subsequent hierarchical levels, which consisted of 421 and 3,514 categories respectively. Filtering out categories with 0 or only 1 human gene in them resulted in 283 and 1,711 categories respectively. One-way ANOVA test resulted with a p-value = 2×10^−16^ for the second level and a p-value = 2×10^−16^ for the third.

**Table 1:** Top categories in Biological Process (GO:0008150) according to Gene Ontology classification system.

GO number	Category name	Number of genes
GO:0000003	reproduction	1169
GO:0001906	cell killing	64
GO:0002376	immune system process	1546
GO:0006791	sulfur utilization	0
GO:0006794	phosphorus utilization	0
GO:0008152	metabolic process	8662
GO:0008283	cell proliferation	1360
GO:0009758	carbohydrate utilization	2
GO:0009987	cellular process	12145
GO:0015976	carbon utilization	0
GO:0016032	viral reproduction	432
GO:0016265	death	1573
GO:0019740	nitrogen utilization	1
GO:0022414	reproductive process	1165
GO:0022610	biological adhesion	884
GO:0023052	signaling	4174
GO:0032501	multicellular organismal process	5182
GO:0032502	developmental process	4094
GO:0040007	growth	705
GO:0040011	locomotion	1112
GO:0043473	pigmentation	52
GO:0048511	rhythmic process	187
GO:0048518	positive regulation of biological process	2973
GO:0048519	negative regulation of biological process	2710
GO:0050789	regulation of biological process	7611
GO:0050896	response to stimulus	5982
GO:0051179	localization	3911
GO:0051234	establishment of localization	3253
GO:0051704	multi-organism process	963
GO:0065007	biological regulation	8045
GO:0071840	cellular component organization or biogenesis	3755

### Correction of the Bias

We evaluated five methods for the correction of the bias. Two of them, VEGAS [9] and GATES [10], has been recently proposed to obtain gene-level statistical significance in GWAS. Although the original goal of both methods is to improve the power of detecting disease associated genes, such approach in principle should also correct, at least partially, the gene size bias in SNP based tests. We also included two simple methods in the comparison, Fisher’s combined probability test [11], and Simes test [12]. Both methods assume that the tests at individual SNPs are independent. In addition, we also proposed a simple method to correct for the size bias. For each gene we suggest adjusting the significance measure by 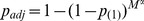, where *p*_(1)_ is the smallest p-value of *M* SNP markers in a gene, and α is a tuning parameter that accounts for the degree of non-independence among the SNPs. We call this method First Order Statistic Correction (FOSCO). Note that this method does not consider a local LD structures of each gene, as GATES and VEGAS do, and adjusts the statistical significance only based on the number of SNP markers per gene, thus ignoring the difference of LD structure between the two sets of SNP markers. Hence, FOSCO is not an alternative to the aforementioned methods in obtaining gene-level significance for individual genes.

To evaluate aforementioned methods, we make use of the data of a GWAS on Schizophrenia [13]. The study was conducted on 2,548 European ancestry subjects (1,170 cases and 1,378 controls). Genotyping was performed using Affymetrix 6.0 array and genotyped by the Birdseed calling algorithm. Association tests are performed on 729,454 SNPs. These SNPs are obtained after filtering out low polymorphic and low quality SNP marker’s sample call rate >97%; SNP MAF <0.01; SNP call rate >0.95; HWE p-value >10^−7^, the criteria described in the original study (http://www.ncbi.nlm.nih.gov/sites/entrez?db=gap; Study Accession: phs000021.v3.p2). All five methods are evaluated on 10 random permutations of the disease status. For FOSCO, the tuning parameter α is determined from an additional random permutation and the value 0.84 is used for other 10 permutations.

For each method, after obtaining (gene-level) p-values, we fit a linear regression model on the logarithm of those p-values with the number of SNP markers of the genes as the explanatory variable. If the gene-size bias is well corrected, we expect the regression coefficients to normally distribute around zero, and the corresponding p-values to uniformly distribute on [0,1].

**Figure 2:**
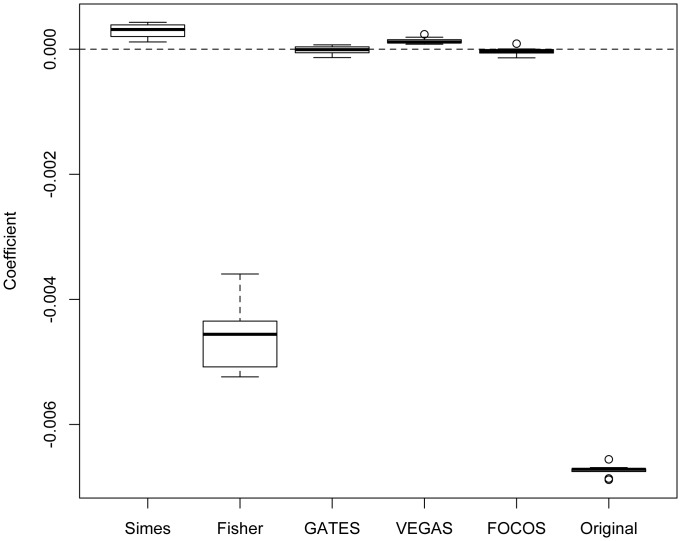
Box-plots of regression coefficients from 10 simulated data with random disease status. The regression coefficients are obtained by regressing the log of gene-level significance p-value on the number of markers per gene.

**Figure 3:**
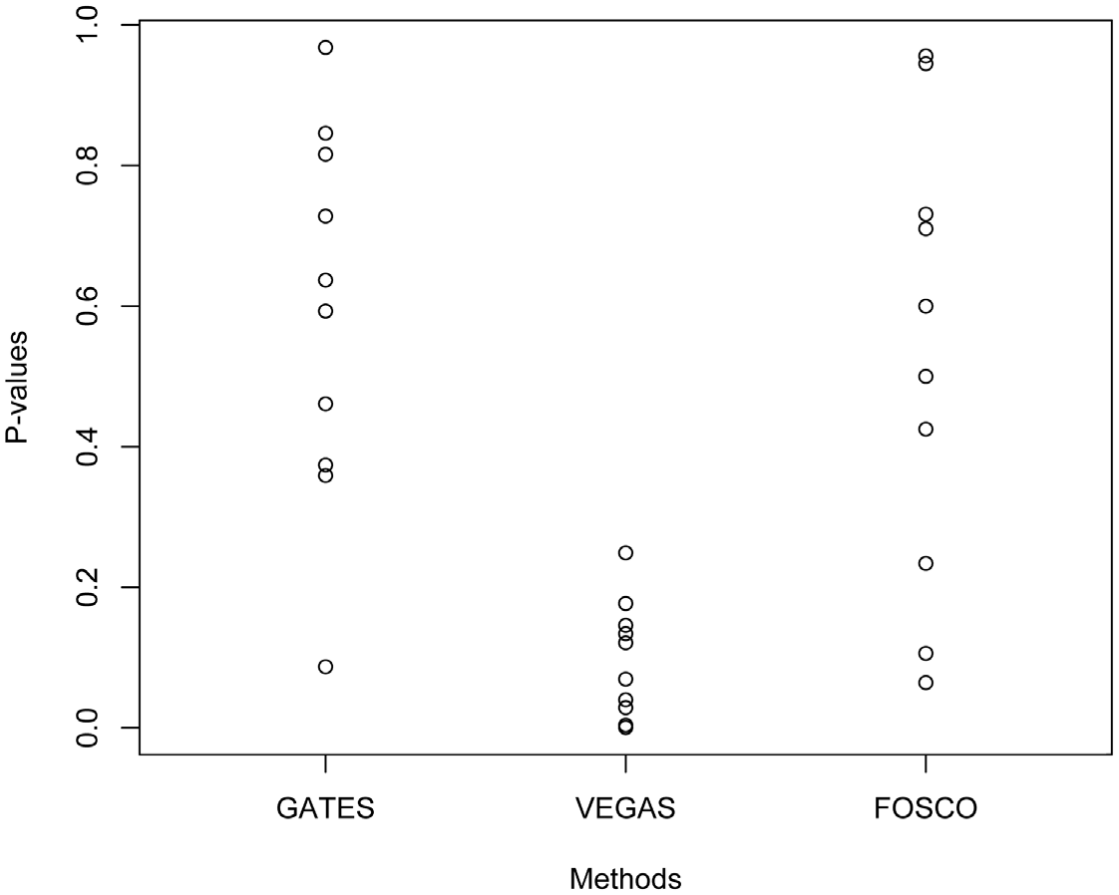
P-values for the linear regression model that regress the log of gene-level significance p-value on the number of markers per gene. Plotted are the results from 10 simulated data with random disease status.

Figures 2 and 3 show the coefficients and p-values from the linear regressions respectively. As one can see in Figure 2, SIMES overcorrected for the size bias, as the coefficients are consistently well above 0; On the other hand, Fisher’s Independent test did a poor job of correcting the size bias. Both GATES and FOSCO have coefficients center around zero and the p-values uniformly distributed from [0,1], showing that the gene-size bias is well corrected. The performance of VEGAS is quite interesting, as the coefficients are all slightly above zero, but p-value not showing statistical significant biases.

## Discussion

The motivation of the study is to demonstrate that there is a strong bias towards favoring genes of large size among those reported as disease related by GWASs. This bias, as we reasoned, is largely caused by the popular method of reporting genes that are closest to the SNP markers with the smallest p-values, without taking consideration of the fact that the number of SNP markers varies a lot among genes. We also demonstrate that the average sizes of the genes among the different gene functional groups are also different, if biased selection of genes are used for downstream *in vitro* and/or *in silico* gene-level studies, the validity of such studies would also be questionable.

Therefore, we propose here that the gene-level statistical significance should be used in the process of selecting genes for downstream gene-level analysis. We examined two recently proposed methods GATES and VEGAS, originally developed to improve the power of genetic association study, to see if the size bias would also be well corrected. We also proposed a simple method for correcting the bias based on the first order statistic. We evaluated these methods on simulated phenotypes using real genotype data from a GWAS study on schizophrenia.

We show that the gene-size bias is well corrected by both our method and GATES, and much reduced by VEGAS. Both GATES and VEGAS were proposed as a method to increase the statistical power of detecting genes associated with the phenotypes, but they also successfully correct the gene-size biases. Here we highly recommend such gene-level significance tests be used for the purpose of prioritizing genes for gene-level downstream analysis. However, the current version of GATES and VEGAS restrict their analysis on specific sets of gene units predefined by their respective developers. For users who wish to work on gene units with their own definition, our simple method provides the flexibility.

## Materials and Methods

### Biases in the Reported GWAS

List of trait related genes (TRG) was obtained from *A Catalog of Published Genome-Wide Association Studies* (http://www.genome.gov/gwastudies) [6]. Number of SNPs per gene was obtained from the NCBI dbSNP (build 131) [7]. Data processing and statistical tests were performed using PERL, MATLAB and R.

### Gene Size Differences Among Gene Sets

Lists of unique genes associated with different biological categories were obtained from “Biological Process” class of Gene Ontology database. Out of 31 categories of the highest hierarchical level 28 categories containing human genes were retained for analysis. The “size” of a gene was determined as number of SNPs associated with the gene according to NCBI dbSNP. One-way ANOVA test was utilized to detect significant difference between average gene size in categories.

### Correction of the Bias

For a gene, let *m* be the number of SNPs in a gene and *p_(i)_* be the p-value of the *ith* SNP.

1. Fisher combined probability test: The p-value is determined based on 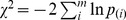, where the degree of freedom of the χ^2^ statistic under the null hypothesis is 2 m.
2. Simes test: 
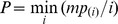
;
3. GATES: 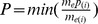, where *m_e_* is the effective number of independent p-values among the *m* SNPs and *m_e(i)_* is the effective number of independent p-values among the top *i* SNPs. *m_e_* is approximated by eigenvalues of the correlation matrix of p-values of m SNP based tests, in which the pairwise correlation between two tests can be approximated by an empirically determined formula given the allelic correlation coefficients between the two SNPs. In the simulation study reported here, the computation is performed on the LD structure derived from CEU subjects of HapMap Project, as this population matches the population used in the Schizophrenia GWAS.
4. VEGAS: for each gene, the reference distribution of the test statistic, sum of Chi-Square statistics over all SNPs, is generated using Monte Carlo simulation taking into consideration the LD among those SNPs. In the simulation study reported here, the computation is performed on the LD structure derived from CEU subjects of HapMap Project.
5. FOSCO: For each gene, the significance measure is given by 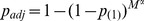, where *p_(1)_* is the smallest p-value of *M* SNP markers in a gene, and α is a tuning parameter. When α = 1, *p_adj_* is the probability of observing the smallest value, i.e. the first order statistic, of *M* independent random variables, all uniformly distributed from [0,1]. The value of α is determined empirically on the data set in which case/control status of subjects is randomly permuted, but the genotypes remain the same. We use a grid search to find the value of α that minimize the absolute value of the correlation coefficient between *p_adj_*’s and number of SNP markers of a gene, and used it as the tuning parameter.

We assign SNPs to genes according to NCBI dbSNP, and use this definition for FOSCO, SIMES, Fisher’s methods. GATES uses the same database for its set of genes but also includes 5 kb flanking regions for each gene. VEGAS uses UCSG Genome Brower hg18 version and include 50 kb flanking regions. Major features of all methods are summarized in Table 2.

**Table 2:** Summary of the methods.

	Simes test	Fisher test	GATES	VEGAS	FOSCO
**Source**	[12]	[11]	[10]	[9]	–
**Core idea**	Adjust p-values by 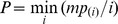 under assumption of independence	Obtain p-value based on 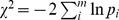 under the assumption of independence	Adjust p-values by 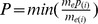 , where me_(i)_ is obtained from each gene empirically	Obtain p-values base on 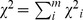 _._ The distribution under the null hypothesis is obtained through Monte Carlo methods	Adjust p-values by 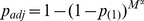 where α is determined empirically from a random permutation
Linear regression coefficient after correction*	2.32E-04	−8.32E-03	−3.14E-04	−1.00E-04	−3.75E-04
**SNPs association** **to genes**	NCBI dbSNPs	NCBI dbSNPs	NCBI dbSNPs+5kb flanking regions	UCSG Genome Brower hg18+50 kb flanking regions	NCBI dbSNPs

Note: * Linear regression coefficient before correction β = −7.23E-03.

### Schizophrenia GWAS Data

We obtained the data from *The NCBI Genotypes and Phenotypes database* (dbGaP; http://www.ncbi.nlm.nih.gov/sites/entrez?db=gap; Study Accession: phs000021.v3.p2) [13]. p-values of SNPs were calculated in the same manner as in the original study (Pearson Chi-Squared test without Yates continuity correction).
